# New Insights Into Pyridoxal Kinase Inhibitors and Their Antileukemic Effects

**DOI:** 10.7759/cureus.48176

**Published:** 2023-11-02

**Authors:** Pallabi Banerjee, Tripti Singh, Imteyaz Qamar

**Affiliations:** 1 School of Biotechnology, Gautam Buddha University, Greater Noida, IND

**Keywords:** apoptosis, flow cytometry, cytotoxicity assays, molecular docking, pyridoxal kinase

## Abstract

Pyridoxal kinase (PDXK) plays a pivotal role as an essential enzyme in cellular processes. It catalyzes the phosphorylation of pyridoxal, pyridoxamine, and pyridoxine to generate pyridoxal 5'-phosphate (PLP), the bioactive form of vitamin B6. An intriguing link has emerged between elevated expression levels of PDXK and PLP and various types of carcinomas, including leukemia. Leukemic cells have an increased need for vitamin B6 to sustain their survival and rapid growth, highlighting the potential of targeting PDXK-PLP as a promising therapeutic target in cancer treatment. To discover a novel and promising PDXK inhibitor, we conducted a comprehensive screening of compounds derived from both natural sources and drug-like databases. Our approach involved employing structure-based virtual screening and molecular docking techniques to attenuate the phosphorylation of PLP. Among the top six compounds, ZINC095099376 (referred to as C03) emerged as the most potent inhibitor of PDXK, primarily due to its exceptional binding affinity and remarkable specificity for the target protein. Furthermore, our investigation revealed that compound C03 establishes crucial interactions with key residues within the substrate binding site, indicating that it binds at the same site as the co-crystallized ligand. Remarkably, compound C03 inhibited the endogenous PDXK expression, showed anti-proliferative activity, and triggered an intrinsic pathway for apoptosis via the activation of key apoptotic factors in leukemic cells. In summary, these findings strongly indicate that compound C03 holds promise as a novel inhibitor of PDXK, offering the potential for the development of effective treatments for leukemia.

## Introduction

Leukemia is a type of cancer that originates from blood cells, specifically from cells that would typically grow into various kinds of blood cells. White blood cells usually develop leukemia in the early stages, but other types of blood cells can also be affected. The disease is categorized into different forms based on its progression rate, such as acute (fast-developing) or chronic (slow-developing), as well as whether it originates from myeloid or lymphoid cells [1]. According to the World Cancer Statistics Report for 2020, leukemia is the fifteenth and eleventh most frequent cause of cancer incidence and cancer-related mortality, accounting for 474,519 incidences and 311,594 deaths, respectively [2]. The majority of cancer cells exhibit metabolic changes linked to tumorigenesis, though it is unclear whether these changes are the source or an effect of cancer phenotypes [3,4].

Living beings are composed of a variety of cells that carry out various metabolic processes to ensure the survival of an organism. The metabolic processes are performed by different enzymes that catalyze the various reactions. These enzymes are function-specific and are proteinaceous in nature. These proteins are produced with the help of the translation of different enzyme-coding genes. One such enzyme is pyridoxal kinase, a vital enzyme in humans encoded by the PDXK gene [5], which is responsible for catalyzing the reaction involving the phosphorylation of pyridoxine (vitamin B6), which leads to the formation of pyridoxine 5’-phosphate [6].

ATP + pyridoxine = ADP + H+ + pyridoxine 5'-phosphate

The enzyme is named after its physiological function, which involves the phosphorylation of pyridoxine. It belongs to the transferases family of enzymes utilizing the alcohol group as an acceptor. The enzyme plays a major role in the synthesis of pyridoxal 5’-phosphate (PLP), pyridoxine 5’-phosphate (PNP), and pyridoxamine 5’-phosphate (PMP) from the dietary vitamin B6 vitamers pyridoxal (PL), pyridoxine (PN), and pyridoxamine (PM). PLP is the active coenzyme form of vitamin B6 that acts as a vital cofactor in over 140 enzymatic reactions. These reactions involve the biosynthesis and catabolism of amino acids and nucleic acids, as well as the regulation of glucose, sphingolipid, and fatty acid metabolism [7,8]. The protein resides in the cytoplasm and functions as a homodimer. Additionally, multiple alternatively spliced variants of it have been identified that also have a biological significance [9]. Lainé-Cessac P, Cailleux A, and Allain P reported the mechanisms behind the inhibition of pyridoxal kinase by several drugs [10]. A report published in 2006 by Martin K. Safo and colleagues presented the crystal structure of PdxY, which is a protein in Escherichia coli that is homologous to PDXK [11]. At the same time, researchers also determined the three-dimensional structures of sheep brain PDXK both alone and in combination with different ligands such as PDXK/ATP, PDXK/AMP-PCP/pyridoxamine, PDXK/ADP/PLP, and PDXK/ADP. These findings have enhanced our knowledge of PDXK’s catalytic mechanism [12,13].

As vitamin B6 may increase cancer risk rather than having a chemopreventive effect, recent studies have suggested that vitamin B6 metabolism significantly contributes to cancer cell proliferation [14]. However, it is difficult to infer metabolic dependencies from genomic analysis alone, so a CRISPR/Cas9 functional genomic screen was performed by Chen et al., revealing that PDXK and the vitamin B6 pathway are selectively dependent in acute myeloid leukemia [15]. Leukemic cells require PDXK kinase activity and vitamin B6 for proliferation while disrupting PLP-dependent metabolism results in decreased levels of nucleotide and polyamine. As leukemic cells heavily rely on the vitamin B6-dependent metabolism for multiple pathways essential for survival, blocking either the PDXK or PLP level has shown antileukemic effects. Therefore, it is possible that vitamin B6 pathway inhibitors might be promising antileukemia drugs by simultaneously inhibiting these pathways [16].

Since most inhibitors bind to the ATP-binding sites of kinases and other cellular enzymes, the development of therapeutic drugs that target PDXK is a challenging task. In order to interrupt PDXK's interactions with PLP specifically, we developed selective and less toxic potential inhibitors that could specifically bind to the regions overlapping with PDXK’s ATP-binding sites. By screening a large number of compounds using structure-based virtual screening, researchers can identify lead compounds that have a high probability of binding to the target protein and can be further developed into potential drug candidates. Therefore, the current study aimed to identify potential inhibitors of PDXK through a high-throughput structure-based virtual screening and molecular docking approach. The novel lead compounds were evaluated and selected by their docking scores and binding affinity, followed by testing on leukemic cell lines in vitro. To assess the efficacy of the compounds, cytotoxicity assays, cell cycle analysis, and Western blotting were performed while qRTPCR was done to assess the apoptotic mechanism. Our study provides new insight into the development of novel and potent anti-cancer drugs targeting the PDXK-PLP complex with minimal side effects using in-silico molecular docking and in-vitro cell-based studies.

## Materials and methods

The three-dimensional crystal structure of the PDXK-PLP complex protein was retrieved from RCSB PDB (Protein Data Bank; www.rcsb.org) with PDB ID: 3KEU [16] and was then further prepared using the graphical user interface (GUI)-based “Auto-Dock Tools” for the docking studies. For this study, we chose to use the Natural and Drug-like compound library of the Zinc database [17] where we downloaded a total of 7,28,747 compounds in two-dimensional structured data file (SDF) format. To prepare these structures (ZINC compounds) for molecular docking studies, we converted them to PDBQT format using Open Babel 2.3.2 [18].

Molecular docking-based virtual screening

Structure-based virtual screening is a computational method used to identify potential hits from a large chemical library by predicting how well a small molecule (ligand) binds to a target protein or enzyme (receptor) based on its three-dimensional structure [19]. This method involves the use of molecular docking programs that simulate the binding process between the ligand and receptor and calculate a score based on the predicted binding affinity [20]. The stability of the resulting complex depends on the number of favorable interactions, such as hydrogen bonds and electrostatic interactions, between the ligands and the receptor. These interactions contribute to the binding affinity of the compounds toward the receptor and can help identify potential hits for further optimization. To determine the binding affinity and conformational pose of compounds to the target protein PDXK, molecular docking was performed using AutoDock Vina (Molecular Graphics Lab, The Scripps Research Institute, USA), a free and open-source software, widely used to predict the preferred orientation of drug candidates and their binding affinities to a target protein [21]. A temperature of 300 K, pH of 7, solvent concentration of 0.1 M, and electrostatic energy cut-off of 15 Å were used as default parameters during docking calculations in this study. After obtaining the compounds with higher docking scores, all possible docked conformers were generated by splitting them. These conformers were then analyzed using Pymol (DeLano Scientific LLC, USA) and Discovery Studio (Dassault Systemes BIOVIA, USA) to investigate their interactions with PDXK. In addition to docking analysis, the SwissADME Calculation program (http://www.swissadme.ch/index.php) was used to evaluate the drug-likeness rankings of the compounds for improved understanding of compound structure-activity relationships [22]. Detailed docking procedures were described in our previous publication [23].

Molecular dynamics (MD) simulation

Desmond version 5.3 was utilized to conduct molecular dynamics (MD) simulations, employing the inherent OPLS-AA force field. The MD simulations were initiated using specific docking poses obtained from molecular docking experiments, as outlined in the methodology by Banerjee et al. [23]. To evaluate the binding free energy (MM-GBSA), calculations were performed using the final 10 ns of the dynamic simulation trajectory of the ligand-protein complex as described previously [23].

Cell lines and cell culture

Studies were conducted using cancer cell lines, K562 (myeloid leukemia cell line), A549 (adenocarcinoma), Jurkat (T cell leukemia), and HeLa (cervical cancer cells), from American Type Culture Collection (ATCC). Cells were cultured at 37 °C and 5% CO_2_ atmosphere using the RPMI1640 medium (Sera Labs, UK) with 10% fetal bovine serum (Gibco BRL, USA) and 1% penicillin-streptomycin (Sigma-Aldrich, USA). The cells were monitored daily and subcultured twice per week until 80% confluency was attained. Chemicals and reagents were obtained from Sigma-Aldrich (Dorset, UK).

Treatments and cytotoxicity assays

All the hit compounds were commercially procured for further biological activities. In order to obtain a stock solution of 10 mM, all the six test compounds, i.e., C01 (ZINC01612996), C02 (ZINC049841390), C03 (ZINC095099376), C04 (ZINC095098959), C05 (ZINC01482077), and C06 (ZINC03830976) were dissolved in dimethyl sulfoxide (DMSO). The use of DMSO as a solvent helps in dissolving the compounds and ensuring their stability during storage. The stock solution was thereafter aliquoted and stored at −20 °C and utilized within 10 days only.

MTT Assay

The MTT assay was employed to assess the cytotoxic effects of compounds C01-C06 on cancer cell lines (K562, Jurkat, A549, and HeLa) by measuring cell metabolic activity and potential induction of apoptosis or necrosis. Cells were seeded in 24-well plates at 5 × 10^4^ cells/well and treated with varying concentrations of the compounds (0-80 µM) for 48 hours [24]. For the MTT test, 100 μL of cell suspension and 10 μL of 5 mg/mL MTT solution in phosphate buffer were added to each well of a 96-well cell culture plate and incubated at 37°C for 4 hours. After removing the MTT solution, formazan crystals in each well were solubilized with a solution of 50% DMF and 10% SDS, followed by an additional one-hour incubation at 5% CO2. The intensity of the purple formazan crystals was measured at 570 nm using a BioRad iMARK plate reader. The cell control group treated with DMSO was considered 100% cell proliferation, and cytotoxicity was determined by the decrease in optical density (OD) relative to this control (100%) [25]. The experiment was performed in triplicates and repeated at least three times for accuracy and reproducibility, and a concentration-response graph was generated to identify optimal drug dosages for subsequent studies.

Trypan Blue Dye Exclusion Assay

The trypan blue exclusion assay was performed to determine the effect of novel compounds (C01-C06) on cancer cell proliferation. The growth inhibitory activity of the compounds was evaluated by culturing cells in a 24-well plate (Corning, USA) at a density of 0.5 × 10^5 ^cells/ml in 1 ml of complete medium. The cells were then cultured for 48 hours at 37 °C in a 5% CO_2_ incubator until reaching a confluency of at least 70%. Following treatment with various concentrations of compounds (ranging from 0-80 µM), the medium was removed, and the cells were washed twice with PBS following incubation with 0.4% trypan blue stain for 5 minutes. After loading the cells onto the cell-counting chamber, they were observed under a light microscope (Olympus, Japan) to determine whether there were viable (unstained) or dead (blue-stained) cells. The number of viable cells was divided by the total number of cells (stained + unstained) to obtain the percentage of viable cells [26].

Assessment of cell cycle profile using flow cytometry

Compound C03 was chosen for further study because it was discovered to be the most potent in reducing cancer cell proliferation. Therefore, the DNA content analysis was performed by using a flow cytometer. K562 cells were seeded (0.5x10^5^ cells/ml) in six well plates and treated with different concentrations of compound C03 (1, 5, and 10 µM). Cells were harvested after a 24-hour time point, washed with 1X PBS, and fixed in 80% chilled ethanol [27]. Samples were treated with RNase-A (50 μg/ml) (Sigma Aldrich, USA) and incubated at 37°C overnight. Further, cells were stained with propidium iodide (5 μg/ml) (Sigma Aldrich, USA) and cell cycle progression was monitored using a flow cytometer (FACSVerse^TM^ BD Biosciences, USA), with excitation at 488 nm laser and emission at 560/670 nm. Cell distribution across the cell cycle was analyzed with Flowing Software (Version 2.5.1; Turku Bioscience, Turku, Finland) and histograms were plotted. An error bar diagram was used to demonstrate the percentage of cells in each phase of the cell cycle [28].

Western blotting analysis

PDXK expression was detected by Western blotting using a K562 cell extract. Initially, the K562 cells (0.5 x 10^5^ cells/mL) were seeded in a 24-well plate with complete growth media. Subsequently, they were incubated and subjected to 24-hour treatment with test compound C03. To lyse the cells, 10 mM Tris (pH 7.4), 150 mM NaCl, 1 mM EDTA, protease and phosphatase inhibitors, and 1% (v/v) NP40 were used, obtained from Sigma Aldrich, USA. The protein samples were incubated on ice for 10 minutes followed by sonication to disrupt any clumps or aggregates and subjected to centrifugation at 15000g for 10 minutes to eliminate any insoluble cellular debris. The protein extracts were then resolved by SDS polyacrylamide gel (12%) electrophoresis obtained from Invitrogen, transferred to PVDF membrane (GE Healthcare, USA), and blocked overnight with 5% skim milk powder in PBS. The primary antibody was incubated with the membrane for two hours, followed by incubation with a secondary antibody for two hours. To detect PDXK expression, primary antibodies specific to PDXK (anti-PDXK antibody, Sigma HPA030196) and rabbit anti-tubulin antibodies were used. Secondary antibodies were conjugated to anti-mouse peroxidase and anti-rabbit peroxidase. 3, 3'-Diaminobenzidine tetrahydrochloride was used to visualize the protein bands [28]. To ensure equal amounts of protein were loaded in each lane, a tubulin protein signal was used as a loading control.

Quantitative gene expression analysis

K562 cells were treated with compound C03 for 12 and 24 hours, with DMSO as a control. RNeasy Mini Kit (Qiagen, Germany) was used to isolate the mRNA from the cells following the incubation period, and the concentration was measured by spectrophotometry. The extracted mRNA was then subjected to analysis via 1% AGE. cDNA was prepared following the manufacturer's protocol using a cDNA synthesis kit (Qiagen, Germany). The quantitative real-time polymerase chain reaction (qRT-PCR) was carried out using SYBR Green I dye with cDNAs as the amplification template, according to the manufacturer's protocols [29] and in order to normalize the expression of test genes, actin was used as an endogenous control.

Statistical analysis of relative mRNA quantification and protein quantification

The mRNA quantity of apoptotic genes was calculated using the formula 2DCt, where DCt1/4Ct represents the difference between the reference gene cycle threshold (Ct) and the test gene cycle threshold (Ct). For normalization, actin was used as a reference gene. For mRNA, protein expressions, and other analyses, a nonparametric Mann-Whitney test (GraphPad Prism version 5, San Diego, CA) was performed to evaluate the significant differences between experimental groups. The levels of significance were determined using a threshold of p<0.05.

## Results

Structure-based virtual screening of compounds and binding affinity analysis

A commercially available library of 7,28,747 natural and drug-like compounds was virtually screened using a molecular docking approach to target the substrate binding pocket of PDXK. The 3D structures of the prepared compounds were docked into a generated grid to encompass the active site of PDXK, including its residues that interact with PLP. In virtual screening, 246 phytochemicals and 521 ZINC compounds exhibited superior binding affinity compared to the co-crystallized ligand. The binding score due to the re-docking of the co-crystallized ligand (PLP) with PDXK was −6.2 kcal/mol. Those promising hits were selected based on the binding score of PLP. The docking analysis led us to select six compounds that can bind the PDXK substrate pocket with binding affinity ranging from -11.6 to -12.5 kcal mol^−1^, which included hydrogen bond interactions with the critical amino acid residues of the receptor protein. Using Discovery Studio Visualizer, we analyzed the types of interactions between these top six hits and the PDXK binding site. There is a strong correlation between lower binding energy and higher binding efficiency, resulting in augmented inhibition. Table 1 summarizes the docking score and protein-ligand interactions of these six compounds with PDXK.

**Table 1:** The protein-ligand interaction profile and binding energy of the top six screened compounds with the PDXK binding site The listed amino acid abbreviations with their three-letter codes denote Gly-Glycine, Thr-Threonine, Asp-Aspartic acid, Phe-Phenylalanine, Asn-Asparagine, Tyr-Tyrosine, Glu-Glutamic acid, Arg-Arginine, Ser-Serine.

S.No.	Compounds	Popular Name	Hydrogen bond interactions	Nature of compounds	Binding energy (kcal/mol)
1.	Compound C01 (ZINC01612996)	Irinotecan	Gly234, Thr186	Natural compound	-12.2
2.	Compound C02 (ZINC049841390)	benzyl-[(4-hydroxyphenyl) methyl] BLAHheptone	Asp118, Thr233	Plant-derived natural compound	-11.9
3.	Compound C03 (ZINC095099376)	[(2R,3R,4S,5S,6S)-4,5-dihydroxy-2-(hydroxymethyl)-6-[(methyl-methylene-dioxo-BLAHyl) methoxy] tetrahydro	Phe230, Gly117, Asp118, Thr47, Asn150, Tyr127, Glu153, Gly234, Gly232	Plant-derived natural compound	-12.5
4.	Compound C04 (ZINC095098959)	[(1R,3As,5Ar,5Br,7Ar,9S,11Ar,11Br,13Ar,13Br)-3a-hydroxy-1-isopropenyl-5a,5b,8,8,11a-pentamethyl-1,2,	Asp87, Arg86, Ser12, Tyr127, Asp118	Plant-derived natural compound	-11.8
5.	Compound C05 (ZINC01482077)	Gliquidone	Ser12, Thr186, Ser187, Asn150	Synthetic compound	-11.7
6.	Compound C06 (ZINC03830976)	Itraconazole	Ser187, Ser12	Synthetic compound	-11.6
7.	PLP	(4-formyl-5-hydroxy-6-methylpyridin-3-yl) methyl dihydrogen phosphate	Thr47, Asp235, Gly234		-6.2

Molecular docking and interaction studies

**Figure 1:**
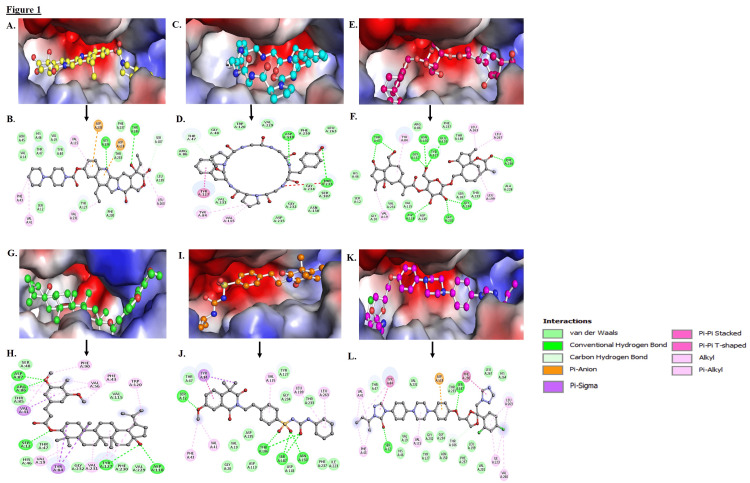
Docked poses and molecular interactions of the selected compounds at the active binding site of PDXK (PDB ID: 3KEU) Three-dimensional hydrophobic surface representation and two-dimensional structural representation of binding pocket residues of PDXK and their interactions with compounds C01(A, B), C02(C, D), C03(E, F), C04(G, H), C05(I, J), C06(K, L). All compounds occupied a similar space in the PDXK binding pocket.

To obtain a theoretical understanding of the potential molecular interactions between the target protein and the screened compounds, molecular docking studies were conducted. These compounds were screened for the best-docked poses and analyzed via PyMol and Discovery Studio Visualizer to determine the receptor-ligand interaction patterns. In Figure 1, well-established good interactions exist between the compounds and the active pocket of the target receptor. The compounds are referred to as C01 to C06, with their corresponding ZINC codes: ZINC01612996 (C01), ZINC049841390 (C02), ZINC095099376 (C03), ZINC095098959 (C04), ZINC01482077 (C05), ZINC03830976 (C06). These studies considered both the ATP binding groove and the PLP interaction sites on PDXK. Figure 1 (A, C, E, G, I, K) illustrates a 3-D surface representation diagram of the protein-ligand interactions, where, all six compounds were found to be deeply bound within the binding groove of PDXK. A detailed analysis of the binding pattern and residues interacting with the top six compounds is shown in Figure 1 (B, D, F, H, J, L). Interaction analyses were conducted to determine the specific non-covalent interactions and their different types. According to the analysis, all the compounds made interactions with the crucial residues (ASP235, THR47, ASN150, THR233) of the PDXK’s binding site and share the same interactions as PLP [23]. In addition to evaluating the physicochemical properties of the compounds that we selected, we also tested their ADMET (absorption, distribution, metabolism, excretion, and toxicity) properties, where all six compounds exhibited good physicochemical properties without any PAINS pattern. The pkCSM program was used to extract various ADMET data, and all six drugs were chosen based on their AMES and hepatotoxicity scores. These six compounds had acceptable ADMET properties and were further investigated as potential PDXK kinase inhibitors since they showed no toxicity (Table 2).

**Table 2:** Calculated ADMET properties of the selected compounds ADMET: absorption, distribution, metabolism, excretion, and toxicity

S.No.	Compounds	Absorption	Distribution	Metabolism	Excretion	Toxicity
		Gastrointestinal (GI) absorption	Blood-brain barrier (BBB) permeability/Central nervous system (CNS) permeation	Cytochrome P450 2D6 (CYP2D6) inhibitor	Organic cation transporter 2 (OCT2) substrate	AMES toxicity
1.	ZINC01612996	High	No	No	No	No
2.	ZINC49841390	Low	No	No	No	No
3.	ZINC95099376	Low	No	No	No	No
4.	ZINC95098959	Low	No	No	No	No
5.	ZINC01482077	Low	No	No	No	No
6.	ZINC03830976	High	No	No	No	No

Molecular dynamics (MD) simulation of the top six docked complexes

**Figure 2:**
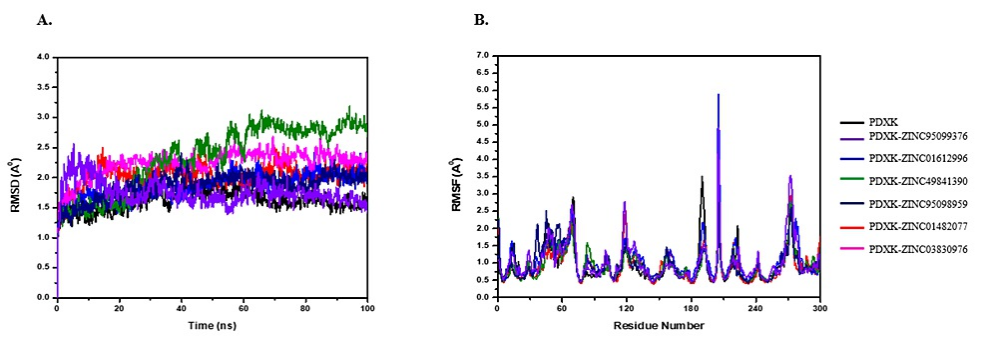
The structural dynamics of the PDXK structure as a function of time (A) RMSD graph showing PDXK in free-state and when bound with compounds. (B) The average residual fluctuations (RMSF) graph of PDXK and upon binding of six compounds. PDXK: pyridoxal kinase; RMSD: root-mean-square deviation; RMSF: root-mean-square fluctuation

To ensure the stability and dynamics of the free protein (PDB Id: 3KEU) and top six ligand-protein complexes necessary for structural changes related to the inhibition mechanism, molecular dynamics simulations of PDXK protein target in complex with top-ranked ZINC compounds (ZINC95099376, ZINC01612996, ZINC49841390, ZINC95098959, ZINC01482077, ZINC03830976) were carried out for the 100 ns time scale using the Schrodinger Desmond package. We evaluated the structural dynamics of PDXK in both its unbound and complex states by calculating the root-mean-square deviation (RMSD) across various time-evolution plots (Figure 2). The fluctuations in the protein backbone were graphically represented using plots generated from the simulations, as illustrated in Figure 2A. The computed average RMSD values for the different systems were as follows: free-PDXK showed 1.62 Å while the PDXK complexes with ZINC01612996, ZINC49841390, ZINC95099376, ZINC95098959, ZINC01482077, and ZINC03830976 exhibited values of 1.86 Å, 2.3 Å, 1.75 Å, 1.85 Å, 2.03 Å, and 2.23 Å, respectively. Notably, compound C03 (PDXK-ZINC95099376) showed a lower RMSD among all the complexes, which indicates greater protein stability. During the simulation time from 10 ns to 50 ns, a modest fluctuation in RMSD, up to 1.0 Å, was observed across all seven PDXK systems. This variance can likely be attributed to the initial positioning of the compound within the PDXK binding pocket.

Conducting RMSF analysis allowed us to investigate residue dynamics within the PDXK structure before and after compound binding. To visualize protein flexibility during MD simulations, we generated RMSF plots for individual residues (Figure 2B). The computed average RMSF values for Free-PDXK and the PDXK complexes (PDXK-ZINC01612996, PDXK-ZINC49841390, PDXK-ZINC95099376, PDXK-ZINC95098959, PDXK-ZINC01482077, and PDXK-ZINC03830976) were 0.92 Å, 1.04 Å, 0.91 Å, 1.02 Å, 0.96 Å, 0.91 Å, and 0.909 Å. The fluctuations in RMSF were notably controlled and minimized upon compound binding, indicating remarkable stability and uniformity within the protein-ligand complex. Multiple peaks indicated flexible amino acids within the protein's C⍺ backbone. The stability and dynamic behavior of these six docked complexes were also evaluated using protein-ligand Rg, H-bond, and SASA calculations, as described in our previous publication [23].

Cytotoxicity assay

**Figure 3:**
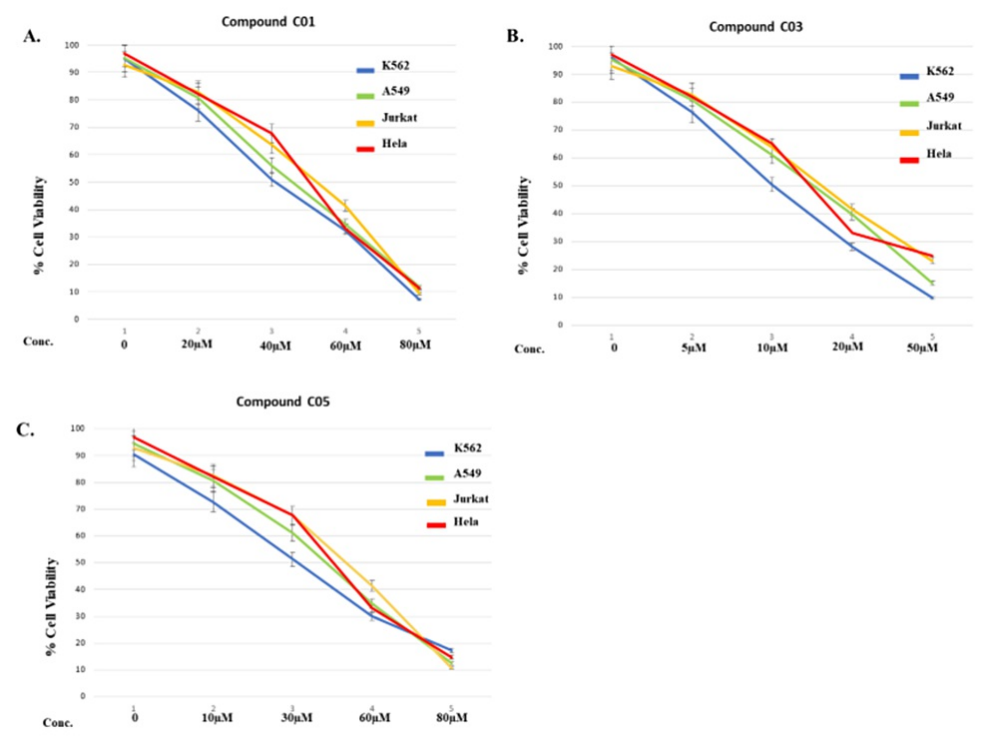
MTT assay to determine the IC50 value of the different drugs and analyze their effect on cell viability after 48 h of incubation The different drug concentrations used and the corresponding cell viability graphs are shown for (A) compound C01 (0 - 80 µM), (B) compound C03 (0 - 50 µM), and (C) compound C05 (0 - 80 µM). The IC_50_ value (that is, the concentration of drug that exhibited 50% cell viability for K562 cells (Blue), A549 (Green), Jurkat (Yellow), and HeLa cells (Red) using an MTT assay at 48h time point) were determined to be 40, 10 and 30 μM, respectively, for C01, C03, C05. Cells without any treatment were used as controls and taken as 100%. y (All data are expressed as mean ± SE from three independent experiments).

The anti-proliferative activity of all six compounds (C01-C06) was evaluated on cancer cell lines, including K562, A549, Jurkat, and HeLa. Using a colorimetric MTT assay, different concentrations of compounds were tested for their effect on cell proliferation after 48 hours of incubation. The results presented in Table 3 indicated that among all the compounds, C03 exhibited the highest inhibition of cell proliferation, followed by C05 and C01. The IC50 values of compound C03 were depicted as 9.97 μM, 11.2 μM, 16.2 μM, and 18.7 μM in K562, A549, Jurkat, and HeLa cells, respectively. Based on the IC50 (<50) values of compounds C01, C03, and C05, the percentage of cell proliferation was calculated in K562 leukemic cells. Interestingly, compound C03 exhibited 50% growth inhibition at a concentration of 10 μM, whereas the other compounds, C01 and C05 exhibited a similar effect at concentrations of 40 μM and 30 μM (Figure 3). Therefore, compound C03 was selected as a lead compound for further biological experiments. Using K562 cells, further experiments were conducted to examine compound C03's mechanism of action.

**Table 3:** Study of the inhibitory effects of the selected compounds: potential lead compounds (C01-C06) have anti-proliferative effects on K562, A549, Jurkat, and HeLa cells IC50 (half-maximal inhibitory concentration) values were determined based on the results of MTT assays conducted at a 48-hour time point.

S.No.	Name of the compounds	Cell lines (IC_50_ Value in μM)			
		K562 (Myeloid leukemia)	A549 (Adenocarcinoma)	Jurkat (T cell leukemia)	Hela (Cervical Cancer)
1.	Compound C01	40.1	44.5	45.06	46.88
2.	Compound C02	> 50	> 50	> 50	> 50
3.	Compound C03	9.97	11.2	16.2	18.7
4.	Compound C04	> 50	> 50	> 50	> 50
5.	Compound C05	30.08	36.6	38.7	41.44
6.	Compound C06	> 50	> 50	> 50	> 50

Novel PDXK inhibitor (C03) exerts an inhibitory effect on cell viability in the K562 cell line

**Figure 4:**
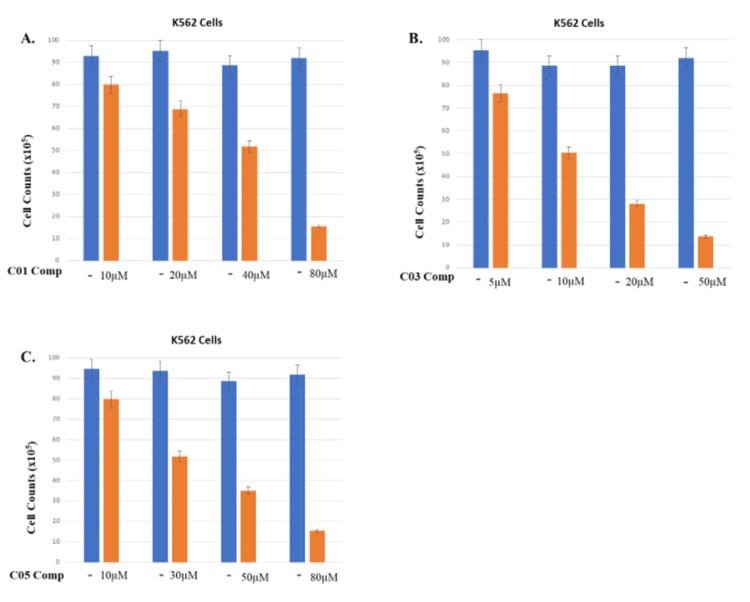
Trypan blue cell viability assay using the K562 cell line After 48 hrs of treatment with increasing concentrations of compounds C01, C02, and C03 as indicated, K562 cells were stained with trypan blue dye for three to five minutes and counted. Cell viability decreased with increasing concentration of compounds and on average, only 50% of cells were viable 48 hours after treatment with various concentrations of compounds.

For further cytotoxicity evaluation, cell viability in the K562 cell line was assessed using the trypan blue assay. Different concentrations of the compounds C01, C03, and C05 were used to treat cells for 48 hours, and the % viable cells were counted. It was observed that compound C03 inhibits cell viability in the K562 cell line at a 10 uM drug concentration, which suggests that C03 has a potent cytotoxic effect on the K562 cells at this concentration (Figure 4). However, further analysis and experiments were conducted to explore the underlying mechanisms and evaluate the potential of C03 as a therapeutic agent.

Novel PDXK inhibitor (C03) resulted in an increased accumulation of cells in the SubG1 phase

**Figure 5:**
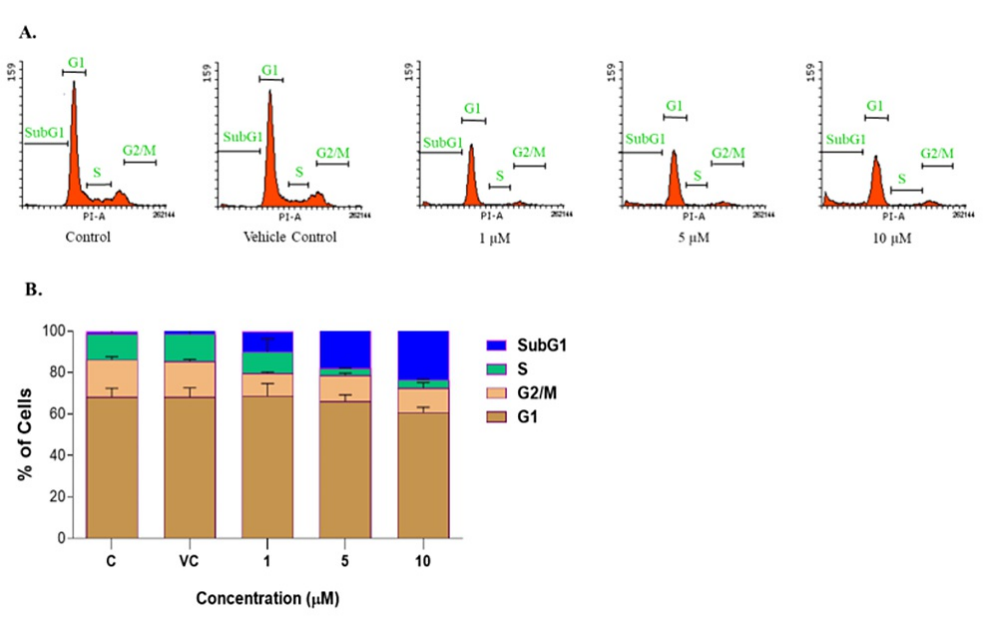
Cell cycle analysis of K562 cells following compound C03 treatment K562 cells were grown in the presence of different concentrations of compound C03 (1,5 and 10 μM) or appropriate concentrations of DMSO (vehicle control) for 12 h. The cells were harvested and stained with ethidium bromide, and the DNA content was quantified by flow cytometry. Histograms showing the percentage of cells in the G0/G1, G2/M, S, and G1 phases of the cell cycle obtained after fluorescence-activated cell sorting (FACS) analysis. For each sample, 10,000 cells were acquired. Each experiment was repeated three times and values are the mean of three replicates ± SE.

After conducting preliminary cytotoxicity assays, subsequent experiments were performed to examine the effect of compound C03 on the cell cycle progression. K562 cells were used to study the DNA content at various stages of the cell cycle (G1, S, G2/M, and SubG1) after treatment with compound C03 at concentrations of 1, 5, and 10 μM. At the 24-hour time point, compound C03 led to a significant change in the cell cycle distribution, particularly in the SubG1 population at a concentration of 10 μM. The percent proportion of different stages of cell cycle phases (histograms) is shown in Figure 5A. The observed increase in the SubG1 population further supports the potential cytotoxic effects of compound C03 on the cell cycle dynamics, indicating that C03-treated K562 cells induce apoptosis and promote cell death in a dose-dependent manner compared to a DMSO-treated vehicle control (Figure 5B).

Novel PDXK inhibitor (C03) downregulates the expression of endogenous PDXK protein

**Figure 6:**
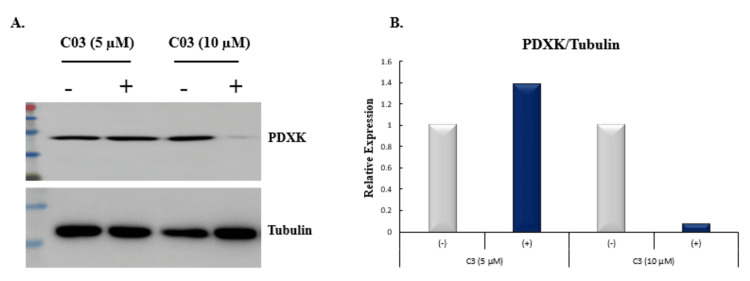
Effects of novel compound C03 on the expression of PDXK in K562 cells. (A) Western blotting analysis after treatment with compound C03 (5, 10 µM) with negative control (DMSO) for 24 h on K562 leukemic cells. Tubulin was used as a loading control. (B) Quantification of PDXK expression relative to tubulin based on western blotting profile presented as a bar diagram (n = 2)

To evaluate the inhibitory action of compound C03 on the expression of PDXK, K562 cells were exposed to two different concentrations (5 μM, 10 μM) of the compound for 24 hours (Figure 6A). After the treatment, the cell-free extracts of K562 cells were prepared and subjected to western blot analysis with tubulin as a loading control. The results demonstrated a significantly low level of PDXK expression after the addition of compound C03 at a concentration of 10 μM. However, at the reduced concentration, the inhibition of PDXK expression was not altered. This result clearly shows that compound C03 acts as a novel inhibitor of PDXK by reducing the endogenous expression of PDXK in K562 cells. The relative expression of PDXK with endogenous control tubulin was also determined using densitometry software (Figure 6B).

Novel PDXK inhibitor (C03) induces apoptosis in K562 cells

**Figure 7:**
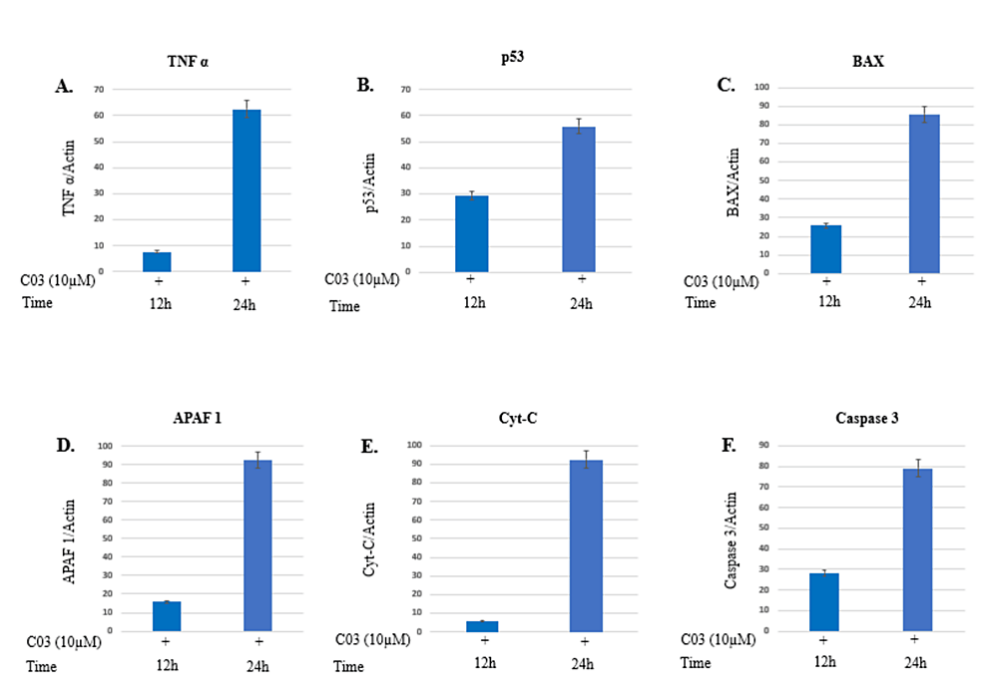
Relative expression of apoptotic factors determined by quantitative real-time PCR (qRT-PCR) analysis (A) Tumor necrosis factor-α (TNF-α), (B) Tumor suppressor (P53), (C) Apoptosis regulator (BAX), (D) Apoptotic protease activating factor 1 (APAF 1), (E) Cytochrome-c, and (F) Caspase 3 genes in K562 cells treated with 10 μm compound C03 for 12 h. mRNA levels of apoptotic factors were determined relative to the endogenous control Actin, according to the formula 2 to the power of delta cycle threshold (2DCt), where DCt¼Ct, reference gene – Ct, test gene. Differences between experimental groups were tested for significance using the nonparametric Mann–Whitney test (GraphPad Prism version 5, San Diego, CA), for both mRNA, protein expressions, and other analyses. Levels of significance are indicated by p< 0.05.

To investigate the apoptotic mechanisms induced by compound C03 on K562 cells, important genes associated with apoptosis activation (listed in Table 4) were analyzed through qRT-PCR. The K562 cells were subjected to treatment with compound 03 at a concentration of 10 μM at two distinct time intervals (12 h and 24 h). The effect of C03 on apoptosis was especially strong in K562 cells upon 24 hours of treatment with a 10 μM dose. After 24 hours of treatment with a 10 μM concentration of C03, K562 cells showed a significant upregulation (4-fold) in the intrinsic apoptotic pathway regulator gene BAX. This overexpression of BAX led to the release of Cyt-C from mitochondrial membranes, which was increased by 8-fold. It was also discovered that the expression of APAF 1 and activated caspase 3 had increased five-fold and eight-fold respectively, at a similar time point (24 hours) following treatment with a 10 μM concentration of C03 [28,30]. In addition, the levels of tumor necrosis factor-alpha (6-fold) and tumor suppressor P53 (2-fold), were also observed to be upregulated upon treatment with 10 μM of compound C03 for 24 hours (Figure 7). Therefore, it can be inferred that the intrinsic pathway of apoptosis is activated in K562 cells treated with 10 μM of compound 03.

**Table 4:** List of Primers used for qRT-PCR qRT-PCR: quantitative real-time polymerase chain reaction

S. No.	Pro-apoptotic – apoptotic genes		
1.	TNF-α	Forward Primer	5′ AGGCGCTCCCCAAGAAGACA 3′
		Reverse Primer	5′ TCCTTGGCAAAACTGCACCT 3′
2.	P53	Forward Primer	5′ CACGAGCGCTGCTCAGATAGC 3′
		Reverse Primer	5′ ACAGGCACAAACACGCACAAA 3
3.	BAX	Forward Primer	5′ TTCATCCAGGATCGAGCAGA 3′
		Reverse Primer	5′ GCAAAGTAGAAGGCAACG 3′
4.	Cyt-C	Forward Primer	5′ AGTGGCTAGAGTGGTCATTCATTTACA 3′
		Reverse Primer	5′ TCATGATCTGAATTCTGGTGTATGAGA 3′
5.	APAF-1	Forward Primer	5′ GATATGGAATGTCTCAGATGGCC 3′
		Reverse Primer	5′ GGTCTGTGAGGACTCCCCA 3′
6.	Caspase 3	Forward Primer	5′ GGTATTGAGACAGACAGTGG 3′
		Reverse Primer	5′ CATGGGATCTGTTTCTTTGC 3′

## Discussion

Resistance to current leukemic therapies is a major challenge in the treatment of leukemia and other types of cancer. To overcome resistance and improve treatment outcomes, there is a need for the development of new chemotherapeutic interventions that can target cancer cells more effectively with fewer side effects. This requires a better understanding of the underlying mechanisms of resistance and the identification of new targets for drug development. One of the recently identified leukemia targets is PDXK [15]. Pyridoxal kinase (PDXK) is an enzyme that plays a critical role in the metabolism of vitamin B6, which is essential for various biological processes, including DNA synthesis, immune function, and neurotransmitter synthesis. According to several studies, PDXK may suppress tumors in a variety of cancers and downregulation of PDXK expression may contribute to tumor growth and progression [15]. Therefore, targeting PDXK with small molecule inhibitors or other therapeutic approaches may represent a promising strategy for cancer treatment. However, further preclinical and clinical studies are needed to evaluate the safety and efficacy of PDXK inhibitors and other targeted therapies for the treatment of leukemia. This study utilized a high-throughput structure-based virtual screening method and molecular docking approach to evaluate the binding efficacy of new lead compounds toward PDXK. Primarily, all six compounds were evaluated as potent inhibitors of PDXK based on their binding affinity and interaction studies. The findings indicated that all the selected compounds exhibited a binding mode similar to the crystal ligand observed in the PDXK-PLP complex (PDB ID: 3KEU). The docking analysis results indicated that the lead compounds interact with PDXK through a shared set of amino acid residues and demonstrated high binding affinity to the substrate binding core having a binding energy between −12.5 Kcal/mol and -11.6 Kcal/mol (C01- C06), which was higher than that of the co-crystallized ligand (PLP) with a binding affinity of −6.2 Kcal/mol. It may also be inferred from the interaction analysis that for designing potent and selective inhibitors of PDXK to specifically interrupt both ATP binding and PDXK-PLP complex formation, it is necessary to target ASN150, THR233, THR47, and ASP235 residues as well as common interactions with ATP binding sites. In addition, the ADME evaluation of the top six compounds exhibited a series of chemical characteristics that may enhance its drug-like properties, and molecular dynamics simulation studies were conducted to validate the stability of the compounds' binding to PDXK [23]. Therefore, it can be concluded from our In silico work that all the six compounds chosen for further study have a higher affinity for PDXK.

In order to investigate the efficacy and mechanism of action of lead compounds, cytotoxic evaluation, cell cycle analysis, western blotting, and qRT-PCR were performed. Among the top six compounds, compound C03 with minimal IC50 of 9.97μM in K562 cells (myeloid leukemia), downregulates the expression of endogenous PDXK in a dose-dependent manner in K562 leukemic cells. Moreover, compound C03 shows a significant accumulation of cells in the SubG1 phase of the cell cycle, and activates key apoptotic factors in K562 leukemic cells triggering the intrinsic apoptosis signaling pathway. In a preceding study, we have made a substantial impact on the realm of cancer research by designing inhibitors targeted at the specific site of PDXK [23]. Altogether, our findings provide compelling evidence for the utility of compound C03 as an effective inhibitor of PDXK and highlight its potential for advancing the development of targeted therapies against cancer.

The study's implications are significant, suggesting that compound C03 holds promise as an inhibitor of PDXK, potentially offering a targeted therapy for leukemia. It highlights the importance of PDXK as a therapeutic target in cancer treatment and provides insights into the compound's mechanism of action. However, limitations include the reliance on silico predictions and in vitro experiments, which may not fully represent real-world complexities. Specificity, off-target effects, and long-term safety must be further evaluated. The study's findings are based on a specific cell line, and clinical translation to humans requires careful assessment. Additionally, ethical, regulatory, and financial constraints may impact future development and may influence the broader research landscape. These limitations emphasize the need for cautious optimism and further research validation.

## Conclusions

In conclusion, the resistance encountered in current leukemic therapies presents a formidable obstacle in the battle against leukemia and other cancer types. To address this challenge and enhance treatment outcomes, the development of novel chemotherapeutic interventions is imperative. The identification of PDXK as a potential leukemia target holds promise, as this enzyme plays a pivotal role in the progression of cancers.

Our study employed advanced structure-based virtual screening to assess newly discovered lead compounds' binding to PDXK. All six compounds displayed strong binding affinity, mimicking the crystal ligand's interaction with PDXK. They exhibited significant binding energy, suggesting potent PDXK inhibition potential. Targeting specific amino acid residues, including ASN150, THR233, THR47, and ASP235, and interactions with ATP binding sites, could facilitate the design of selective PDXK inhibitors. Additionally, these compounds showed favorable ADME characteristics, enhancing their drug-like attributes. Compound C03, among the top six, exhibited remarkable cytotoxicity with an IC50 of 9.97 μM in K562 cells. It effectively downregulated PDXK expression in a dose-dependent manner, arrested the cell cycle at the SubG1 phase, and activated apoptotic factors, triggering the intrinsic apoptosis pathway. In summary, in silico and in vitro findings strongly support the potential of compound C03 as a promising inhibitor of PDXK. This research offers valuable insights into the development of targeted therapies for leukemia and underscores the significance of PDXK as a therapeutic target in the fight against cancer. These results open new avenues for advancing the field of cancer research and the development of effective treatments. Further preclinical and clinical studies are warranted to validate the safety and efficacy of PDXK inhibitors and other targeted therapies in the treatment of leukemia and other cancers.
